# Spatial and temporal activity patterns of *Amblyomma americanum*

**DOI:** 10.1186/s13071-025-06661-x

**Published:** 2025-01-16

**Authors:** Daniel S. Marshall, Karen C. Poh, Mason V. Reichard, Lindsay A. Starkey, Jeb P. Owen

**Affiliations:** 1 Department of Entomology, Washington State University, 100 Dairy Road, Pullman, WA USA; 2 Animal Disease Research Unit, USDA ARS, Washington State University, Pullman, WA 4015 ADBF USA; 3 Veterinary Pathobiology, College of Veterinary Medicine, Oklahoma State University, 250 McElroy Hall, Stillwater, OK USA

**Keywords:** *Amblyomma americanum*, Behavioral ecology, Dispersal, Host-seeking, Risk assessment

## Abstract

**Background:**

Estimates of tick abundance and distribution are used to determine the risk of tick-host contact. Tick surveys provide estimates of distributions and relative abundance for species that remain stationary and wait for passing hosts (i.e. questing), but measures of tick populations may be less reliable for species that actively move in search of a host, such as *Amblyomma americanum*, the lone star tick (LST). Risk estimates for contact with adult LST require knowledge of the tick's spatial and temporal activity. Understanding the movement and the temporal patterns of host-seeking behavior will enhance risk assessment for LST.

**Methods:**

Using CO_2_-baited traps over a 2-year period, we collected wild adult LST in Oklahoma. We used mark-recapture techniques to determine the distance ticks will travel, the proportion of the tick population that is detectable over time, and the relationship between tick abundance and the number of ticks detected in the field. Using video tracking software, we measured the distance traveled and activity time in the laboratory.

**Results:**

In 24 h, LST travel up to 9 (mean = 3.2, SD = 3.6) m in the field and 36 (mean = 70.4, SD = 81.0) m in the laboratory. Marked LST were detectable in the environment for up to 14 days after release. We found that the number of recaptured ticks significantly increased with the relative abundance of ticks released, and at a minimum abundance (N = 1 tick released) LST were detectable 33.3% of the time. Across all experiments, fewer than half of marked ticks were recovered and at most 28.4% of ticks were detected with CO_2_-baited traps at any given time.

**Conclusions:**

Our results show that LST actively move through the environment and pose a risk for host contact at distances of tens of meters. Ticks are detectable for several weeks, but only a fraction of them are detectable at any time. Larger numbers of ticks are detected as their population size increases, but even at very low numbers, LST are recovered with CO_2_ baiting. These spatial and temporal aspects of LST behavior should be considered when building predictive risk models of LST-host contact.

**Graphical Abstract:**

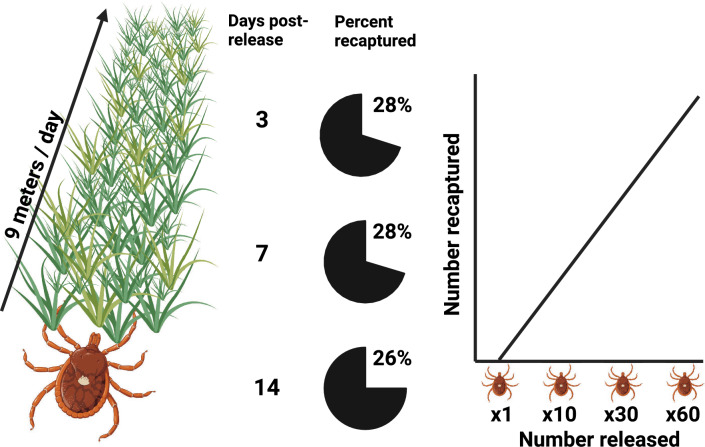

**Supplementary Information:**

The online version contains supplementary material available at 10.1186/s13071-025-06661-x.

## Background

In recent decades, reported tick-borne pathogen infections in the USA have increased [1, 2] with the number of cases reported to the Centers for Disease Control and Prevention more than doubling from 2004 to 2017 [1]. Prevention of tick-borne infections relies, in part, on estimates of tick densities, distributions, and activity patterns, because these variables affect tick-host contact [3–5]. For example, exposure to *Borrelia burgdorferi*, the causative agent of Lyme disease, is strongly correlated with the density of host-seeking *Ixodes* spp. nymphs [4], the location of nymphs along trail edges where people may encounter them [3], and the phenology of host-seeking behavior [6]. The distributions and activity patterns of ticks reflect responses to environmental conditions and behavioral mechanisms that promote host contact [5–8], and the models that best predict tick-borne pathogen risk include environmental variables that affect tick behavior and abundance [5, 7, 8].

Most tick-associated diseases are attributed to hard ticks (Ixodidae) [9] that spend most of the life cycle off the host in the open environment [10]. Tick-host contact is influenced by tick movement in the environment and the timing of host-seeking activity [11–14]. Two generic types of tick host-seeking behavior are “questing” and “hunting” [15–17]. Questing behavior involves the tick climbing vegetation and waiting for contact from a passing host [17, 18]. In contrast, hunting behavior involves active movement across the ground as the tick tracks and orients to host-emitted carbon dioxide (CO_2_) and other cues [15, 16]. Tick sampling methodologies rely on these two foraging strategies when estimating risk of tick-host contact. Dragging or flagging, which involves passing fabric over vegetation, collects questing ticks but misses active hunters which are absent from vegetation [19, 20]. In contrast, CO_2_-baited traps attract ticks from the area surrounding the trap which may or may not have been questing prior to detecting host cues [21]. Although these behaviors may be presented as fixed traits [12, 22, 23], they are representative of two ends of a spectrum with many ticks demonstrating some degree of both behaviors [14, 18, 24–26]. For example, *Ixodes* spp. tend to adopt the questing strategy but will crawl toward stationary hosts that are close by (≤ 50 cm) [14, 27]. In contrast, some ticks in the genus *Amblyomma* hunt their hosts [16, 21, 28] and prefer walking on the ground to climbing vertical structures [29], though they can be found questing on vegetation [24, 25, 30]. Studies of lone star ticks (LST; *Amblyomma americanum*) illustrate the potential for *Amblyomma* spp. to move in the environment. In the wild, LST have been reported moving 5–12 m over a 24-h period [21, 28] and up to 23 m over a 72-h period [31].

Recently, tick range expansion has become a concern regarding the spread of tick-borne pathogens [32–34]. The range of LST has expanded considerably since the 1890s, spreading from the southern US into the Midwest, mid-Atlantic, and southern New England [35–37]. The LST can directly harm livestock [38] and transmit pathogens to people, pets, and livestock. Pathogens transmitted by LST include the causative agents of Rocky Mountain spotted fever [39], Heartland virus disease [40], Bourbon virus disease [41], tularemia [42], and human monocytotropic ehrlichiosis [43], among others. In addition, LST bites are associated with alpha-gal syndrome, an induced allergy to mammalian meat that is occurring in an increasing number of people in the US [44, 45]. Given the health importance of LST and the expansion of the tick’s geographic distribution, it is important to understand the factors that affect the probability of human and animal contact with LST.

Although LST exhibits active hunting behavior [21, 28], the distance the tick can travel in the environment, the amount of time LST remains active in a location, and whether LST density affects the probability of tick contact over time are unclear from earlier studies. These knowledge gaps limit our ability to estimate the risk of LST contact and mitigate tick-borne disease. Predictive models of LST-host contact, activity, and risk for LST-borne pathogen transmission neglect horizontal tick movement [46–49]. Given that LST are highly mobile, current models likely underestimate risk of LST contact. We used a mark-recapture experimental design to determine the spatial range of LST movement toward a host cue in the field. We monitored LST re-captures over time to determine temporal activity patterns of host-seeking ticks. In addition, we determined the effect of tick abundance on the number of ticks encountered (recaptured). Lastly, we observed wild LST movement under laboratory conditions to quantify movement patterns (total distance moved and time spent moving) among individual ticks. These data provide baseline information about the movement capabilities of LST that can be used in future modeling efforts to quantify tick-host contact. We discuss the results in the context of risks for LST-borne pathogen transmission in tick habitat and risk models.

## Methods

### Field site location

Tick collection and mark-recapture experiments were performed at Lake McMurtry recreational area, 13 km northwest of Stillwater, Oklahoma (36.18227, -97.17741). Ticks were collected from a hardwood forest and tallgrass prairie habitat with no recent burn history (previous burns were more than 5 years prior). Mark-recapture experiments took place in hardwood forest of predominantly blackjack oak (*Quercus marilandica*) and post oak (*Quercus stellata*) (see Additional file 1_v2 for maps of the field site and mark-recapture locations)*.*

### Tick collection

Wild adult LST were collected using CO_2_-baited stations from May through July 2023 and March through May 2024. The stations were constructed from 9-quart (8.5-l), 33 × 23 × 20-cm, insulated coolers (Igloo Products Corp., Katy, TX) containing approximately 2.7 kg dry ice. Eight holes (0.635 cm diameter) were drilled through the sides (three holes on long sides and one hole per short side) of each cooler through which CO_2_ sublimed (see Additional file 1_v2 for an image of the tick trap). The stations were placed on the ground in forested areas of Lake McMurtry, and ticks that were attracted to the CO_2_ climbed onto the surfaces of the coolers. Collection sites were selected in areas where ticks had been previously encountered. After 24 h, adult LST were collected from surfaces of the coolers. Host-seeking adult ticks were also collected opportunistically from the surrounding vegetation and our clothing. We visually confirmed that coolers retained dry ice at the end of the 24-h sampling period. Collected ticks were stored at 10 °C ± 2 °C, 90% ± 5% relative humidity, and 14:10 light:dark day length in the laboratory until they could be marked and released or used in laboratory observations. We chose to keep ticks in a cold environment to minimize physiological aging (and any behavioral changes that may accompany it) prior to experimental tests [50]. Ticks were kept for 2–79 days before mark and release (= retention time). To avoid confounding experimental treatments with the time elapsed since tick capture, ticks from multiple retention times were used in each experiment and treatment level.

### Tick marking

Adult LST were randomly selected and restrained using the method described by White et al. [51]. Briefly, painter’s tape was placed on a laboratory counter with the adhesive side facing up. Ticks were placed onto the tape ventral side down. Using POSCA paint markers (Uni Mitsubishi Pencil, Tokyo, Japan) or SmartWater fluorescent taggant (DeterTech, Telford, UK), a small mark was placed in the center of each tick’s dorsal surface so that it did not occlude the eyes or interfere with spiracular plate function. For the distance and time experiments, we first used SmartWater (three experimental cohorts) and subsequently switched to POSCA paint markers (17 experimental cohorts) because it was easier and faster to apply, and it allowed us to apply different color marks. In the abundance experiment, all ticks were marked with POSCA paint markers. We applied different colors to cohorts of ticks to ensure that ticks tested in adjacent plots could be differentiated (see Additional file 1: Figure S1 for an example of a unique marking combination).

To test whether paint marks caused mortality, we painted control ticks in two batches: (1) those collected in 2023 and (2) those collected in 2024. For the 2023 collections (distance, time, and laboratory experiments), N = 20 ticks were painted with POSCA, N = 20 were painted with SmartWater, and N = 23 were unmarked. Ticks were placed into chambers held at 10 °C ± 2 °C and 90% ± 5% relative humidity. A 14:10 light:dark cycle was maintained in all chambers. All ticks were checked daily for mortality for 1 week (the duration of the time recapture experiment). For 2024 collections (abundance experiment), N = 106 ticks were painted with POSCA and placed into chambers kept at 10 °C ± 2 °C and 90% ± 5% relative humidity with a 14:10 light:dark cycle. Mortality was checked at the end of the abundance experiment.

### Distance recapture experiment

To measure distances moved by LST, we placed CO_2_ stations at different distances from the release point of marked ticks. Plots were located in hardwood forest habitat at Lake McMurtry, and we separated plots by a distance of at least 20 m. Prior to releasing ticks, we placed CO_2_ stations at one of seven distances away from the tick release point: 0, 1, 3, 6, 8, 9, or 10 m. Not all distances were tested equally, because our objective was to determine the maximum distance LST travel in a 24-h period. Thus, test distances were adjusted over the experiment relative to the furthest distance traveled in previous replicates. A 0-m control plot was included with every test distance. The 0 m distance was used as a control to confirm that the released ticks were viable and would crawl onto a station.

Movement distances were tested in separate plots, and only one distance was tested per plot. For example, one plot contained stations at the 3 m distance only, whereas a separate plot contained stations at the 9 m distance only. For distances of 1 to 10 m, we placed four CO_2_ stations at cardinal directions away from the tick release point. In the 0 m control plot, we placed one station 0.5 m adjacent to the location of tick release. We expected that one station placed at this distance was sufficient to verify recapture efficacy.

In each replicate plot, we released 30 marked ticks by tipping ticks out of a vial onto a single location of the ground. In each trial, the CO_2_ stations were left in place for 24 h to allow ticks to move towards the stations. After 24 h, ticks were collected from stations. A tick check was performed by field personnel prior to exiting the plot to ensure marked ticks were not transported out of their respective plots. The number of recaptured marked ticks was recorded for each station in a plot. Each plot was sampled a single time, except for 0 m control plots (see time recapture experiment below). Tests of LST movement were conducted from May through July 2023. We obtained daily weather data for all field experiments from the Stillwater Airport Mesonet station located approximately 8 km southeast of our field site [52, 53].

### Time recapture experiment

To evaluate tick recapture over time, we re-deployed CO_2_ stations at multiple timepoints after tick release. For these tests, we used the 0-m control plots from the distance experiments (see previous section), where we placed a single CO_2_ station adjacent to the location of tick release. The 0-m control plots were used to avoid drawing ticks away and confounding effects of spatial dispersal with time. We re-deployed stations at the 0-m location in six plots from May through July 2023, and plots were sampled at 0, 3, and 7 days after marked tick release, except for one plot, which was sampled at 0 and 3 days after release. We recorded the number of marked ticks captured at each sampling timepoint.

### Abundance effect experiment

To test whether recaptures were affected by the abundance of ticks, marked ticks were released in six plots at least 30 m apart at 14, 7, and 3 days prior to collection so that each plot contained groups of ticks released at the three separate timepoints. We released ticks at four relative abundance levels (60, 30, 10, and 1 tick) in each plot. We used a full factorial design such that the relative abundance levels were replicated at each release timepoint (see Additional file 2: Table S1). Thus, each plot contained 103 marked ticks that reflected a mixture of time periods between release and recapture (*N* = 618 total across all plots). Ticks were uniquely marked to distinguish the plot and treatment group (relative abundance + release timepoint). Fourteen days after the release of the first tick groups, a single CO_2_ station was placed in every plot 0.5 m from the location of tick release. Thus, all plots were sampled at the same time, and three time intervals (release to recapture) were represented in each plot. Stations were left in place for 24 h. When releasing marked ticks and deploying CO_2_ stations, personnel wore picaridin insect repellent and self-checked for ticks so that marked ticks were not removed from plots prematurely. Tests of abundance on LST recapture took place during June 2024.

### Laboratory observation experiment

To characterize LST travel distances and activity times in the absence of environmental heterogeneity, we observed wild ticks in laboratory arenas. Wild-caught LST were stored in laboratory incubators held at conditions described in the tick collection section above. Ticks were collected from the field 13 April–1 July 2023. Time intervals between capture and laboratory recording ranged from 0 days to 5 months, and ticks were selected such that similar time intervals (i.e. retention times) were represented for each test. Ticks were placed into observation chambers (glass petri dishes), and behavior was recorded using a digital camera (Canon EOS Rebel T100, Canon Inc., Ōta, Japan) interfaced with a desktop computer. We used petri dishes with bases that nested inside a larger lid to prevent ticks from escaping. Petri dishes were placed on top of an aquarium turned on its side. A ring light with a Mylar filter was placed inside the aquarium to provide bright, diffuse illumination from below the petri dishes. The camera was placed above the petri dishes so that ticks were illuminated from below. This prevented glare from interfering with the tracking software (see Additional file 1: Figure S2 for a photo of the recording apparatus). Care was taken to ensure the focus and field of view were consistent across recordings. We recorded behavior for 24 h in a dark room with no sources of host stimuli held at approximately 24 °C and 60% relative humidity. We recorded ticks for 24 h, uninterrupted, to capture any effects of tick circadian rhythm on behavior and to limit effects of tick handling and set-up. For each trial, we quickly placed a tick into the center of each petri dish using featherweight forceps and then covered the dish with the lid. We treated walking on the petri dish lid the same as walking on the base. Chambers were cleaned prior to each observation using hot water and dish soap and then rinsed with 75% ethyl alcohol. Video recordings were analyzed with EthoVision software (Noldus Information Technology, Wageningen, The Netherlands), and we calculated the total distance moved and time spent moving by each tick.

### Statistical analyses

We used Poisson family logistic generalized linear mixed effects models to analyze the number of recaptured ticks at tested distances and abundances. For the distance experiment, we modeled the number of marked ticks recaptured as a function of distance from LST release point, with date of initial tick capture, experiment date, and plot location as random factors: *Number*_*marked*_ ~ *Distance* + *(1|Date*_*collection*_*)* + *(1|Date*_*experiment*_*)* + *(1|Plot)*. For the abundance experiment, we modeled the number of recaptured ticks as a function of marked tick relative abundance, time since release, and the interaction of relative abundance and time; date of initial tick capture and plot location were treated as random factors: *Number*_*marked*_ ~ *Relative Abundance* + *Time* + Relative Abundance * Time + *(1|Date*_*collection*_*)* + *(1|Plot).* A binomial family generalized linear mixed effect model was used to analyze the recaptured proportion of ticks at days 0, 3, and 7 after release for the time experiment. For the recaptures over time, we calculated the proportion of marked ticks recaptured on day X relative to the number of *uncaptured* ticks in a plot on day X: Recaptured *Proportion *_day X_ = number of ticks recaptured on day X / (30 – number of ticks recaptured on preceding days). We modeled the proportion recaptured as a function of time (Days = days from LST release); date of initial tick capture, experiment date, and plot location were treated as random factors: *Recaptured Proportion *_day X_ ~ *Days* + *(1|Date*_*collection*_*)* + *(1|Date*_*experiment*_*)* + *(1| Plot)*. We used a chi-squared test to compare the numbers of ticks caught at stations in the four cardinal directions of the distance plots. Statistical analyses were carried out in R [54] using the *lme4* (v. 1.1–29) [55] and *rstatix* (v. 0.7.2) [56] packages.

## Results

### Tick marking

The marking procedure had no effect on tick survival in the mark-recapture experiments. No marked or unmarked ticks died over the duration of observation (7 days), and no difference in survival was observed between ticks marked with POSCA paint, those marked with SmartWater, and unmarked ticks.

### Distance recapture experiment

**Fig. 1:**
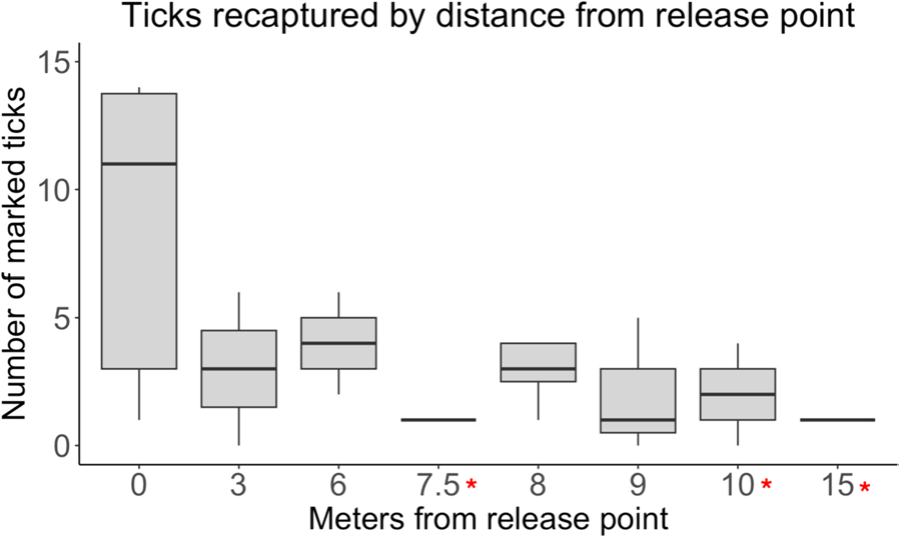
The number of *Amblyomma americanum* adults recaptured decreased with distance from release point. We sampled marked ticks at 0, 3, 6, 8, 9, and 10 m distant from their release point. Black dots represent the number of marked ticks collected in 24 h. Red asterisks represent estimated distances traveled in 24 h from ticks that were collected outside of their plots. Two distances (7.5 m and 15 m) were not sampled in the field but were calculated as distances travelled within 24 h. No ticks were captured at 10 m, but two ticks were calculated to have travelled that distance within 24 h

A total of 600 marked *A. americanum* were released across 20 test plots. Ninety (15%) marked ticks were recaptured across all distances (Table 1). The number of marked ticks collected declined with increasing distance from the point of release, with the most ticks recaptured at the release point, distance zero (*z* = -4.904, *P* < 0.0001) (Fig. 1) (model outputs are provided in Additional file 2: Table S2_v2). Over the 24-h test period, the median (range) number of ticks recovered across all test distances (1, 3, 6, 8, 9, and 10 m) was 3 (0–14) ticks. The greatest distance at which ticks were recovered was 9 m. We collected six marked ticks at two neighboring plots 30 m from their release points between 2 and 19 days after their release. The neighboring plots, which were in use for the time recapture experiment, were sampled two to four times during that period. We infer that ticks were drawn towards neighboring plots during sampling days when CO_2_ stations were present. Based on the linear distances from the original release points to the points of capture in neighboring plots, we estimated the distances traveled by these ticks over 24 h after their release [= (total distance traveled/total hours between release and capture) × 24 h]. The mean (range) estimated distance traveled by these ticks was 10.4 m (7.5 − 15.0 m) (Fig. 1; red asterisks). The ambient field conditions over the course of the distance experiments ranged from 8.86 − 35.24 °C and 30.49 − 99.50% relative humidity. Our model accounted for daily weather conditions by including the experiment date as a random effect (weather data are included in Additional file 3_v2). Ticks that were recaptured at distances > 0 m from the release point were not evenly distributed among the four cardinal direction traps. No plots recaptured ticks in all four traps and 42.9% of plots recaptured ticks in one or two traps. The numbers of ticks recaptured among the four cardinal directions were not statistically different (*X*^*2*^ = 12.988, *df* = 18, *P* = 0.6032), indicating that there was not a uniform directional bias in recaptures among all plots.

**Table 1:** Released and recaptured ticks at experimental distances. The greatest distance at which ticks were recaptured was 9 m. A total of 15% of marked ticks were recaptured across all distances

Distance (m)	Ticks released	*N* recaptured ticks	Percent ticks recaptured
10	30	0	0.00
9	90	6	6.67
8	120	14	11.67
6	90	12	13.33
3	60	6	10.00
1	30	0	0.00
0	180	52	28.89
Total	600	90	15.00

### Time recapture experiment

**Fig. 2:**
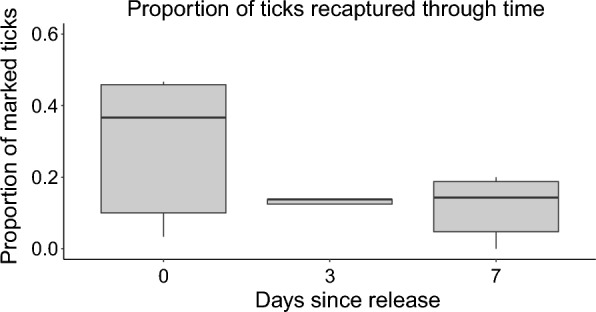
The adjusted proportion of *Amblyomma americanum* adults recaptured through time. Adjusted proportion is the proportion of marked ticks captured of those available at the time of sampling (i.e. the proportion captured out of the number of ticks released minus those captured on previous sampling days). We sampled plots at 0, 3, and 7 days after the release of marked ticks

Among the six plots resampled over time at the 0 m distance (point of release), 77 marked ticks (42.8%) were recaptured between days 0 and 7 (Fig. 2). There was a significant negative relationship between the proportion of marked ticks recaptured and time since release (*z* = -2.418, *P* = 0.0156). The median (range) numbers of ticks caught on days 0, 3, and 7, were 11 (1 − 14), 4 (1 − 6), and 2 (0 − 3) ticks, respectively. The mean (range) adjusted proportions of ticks recaptured on days 0, 3, and 7 were 0.29 (0.03 − 0.47), 0.17 (0.06 − 0.38), and 0.12 (0 − 0.19), respectively. Ambient field conditions ranged from 8.86 − 35.24 °C and 30.49 − 99.50% relative humidity over the course of the time recapture experiment.

### Abundance effect experiment

**Fig. 3:**
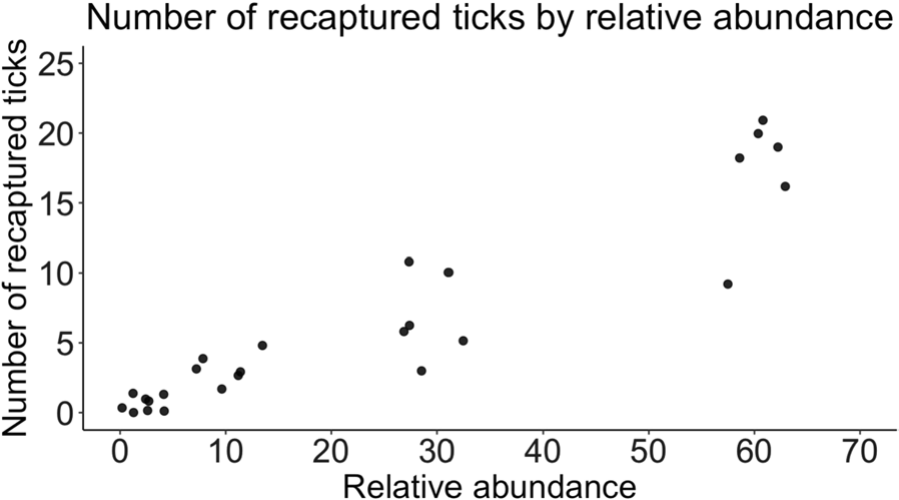
The number of marked *Amblyomma americanum* adults captured across four relative abundances (*N* = 1, 10, 30, and 60 ticks). Points are jittered to better visualize their distribution

We recaptured 170 (27.6%) ticks released in the relative abundance experiment. The total numbers of marked ticks captured from relative abundance groups of 60 (N = 360 ticks), 30 (N = 180 ticks), 10 (N = 60 ticks), and 1 (N = 18 ticks) tick(s) were 103 (28.7%), 41 (22.9%), 20 (33.3%), and 6 (33.3%), respectively. There was a significant positive relationship between the number of recaptured ticks and relative abundance of the released ticks (*z* = 6.651, *P* < 0.0001) (Fig. 3). The total numbers of marked ticks recaptured from release-time treatment groups (*N* = 206 ticks/group) of 14, 7, and 3 days were 54 (26.2%), 58 (28.4%), and 58 (28.2%) ticks, respectively. There was no significant effect of time since release on the number of ticks recaptured (*z* = 0.827, *P* = 0.408). Ambient field conditions ranged from 13.65−36.19 °C and 30.08−99.67% relative humidity during the 2 weeks marked ticks were in the field. A total of 1.2 cm of rain fell over 4 days between the 14- and 7-day tick releases. On two occasions, marked ticks (*N* = 2) were found on the author’s clothing after having released marked ticks into plots. The time and location of their release were identified, and the number of ticks in those plots was reduced accordingly.

### Laboratory observation experiment

**Fig. 4:**
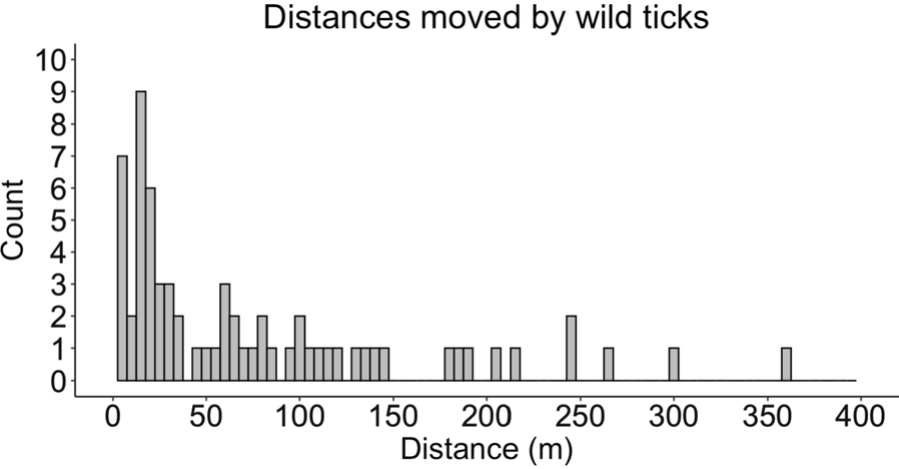
Counts of distances traveled by wild-caught *Amblyomma americanum* in laboratory observations for 24 h

**Fig. 5:**
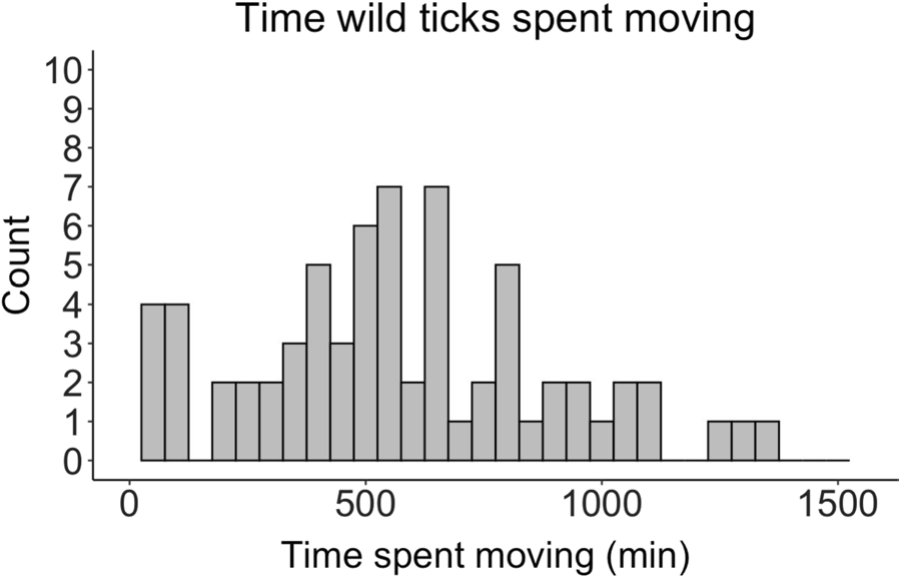
Counts of time spent moving by wild-caught *Amblyomma americanum* in laboratory observations for 24 h

We observed 72 (*N* = 43 female, *N* = 29 male) wild-caught LST under laboratory conditions (Additional file 2: Table S3). Durations of time between capture and observation ranged from 0 days to 5 months. Ticks moved a maximum of 359.9 m and a mean (SD) distance of 70.4 (81.0) m (Fig. 4) and spent a mean (SD) time moving of 542.6 (336.6) min (37.7% of the observation period) (Fig. 5). Not all LST were active in this experiment. We found that 11.1% of observed ticks did not move during the 24-h observation window.

## Discussion

Movement and activity patterns of hard ticks affect their distributions in the environment and the probability of host contact [5–8, 12–14]. We conducted a mark-release-recapture experiment with wild adult LST, *A. americanum*, to estimate the distances LST move, the proportion of the tick population that is detectable over time, and the proportion of the tick population that is detectable at different abundances. In addition, we measured wild LST movement in the laboratory to characterize movement parameters (distance traveled and time spent moving) in a uniform environment. We found that LST were able to move up to 9 m in the field over 24 h, and ticks moved 30 m over a period of 19 days. Spatial complexity in the field likely limited the range of LST movement. In laboratory arenas with no structural complexity, LST were observed to move 40 times farther than what we observed in the field.

Our results align with older studies of LST movement that have shown LST travel 5–21 m in a 1–3 day period after release [57] [28] [21]. This degree of movement is substantially greater than observed with non-*Amblyomma* tick species that travel < 1–2 m over a period of days to weeks [14, 26, 58, 59]. Our recoveries of LST also mirror previous studies that recaptured 4–26% of ticks within 24 h of release [21, 28, 57, 60]. We did not observe a consistent relationship between time post-release and recapture within the timeframe of our experiments. Our data illustrate that only a subset of the total number of ticks in an area were detected by CO_2_ trapping at any given time. Marked ticks that were never recaptured may have crawled out of the sampling plots, attached to hosts, entered quiescence and remained inactive at the site of release, or died. A subset (11.1%) of LST observed in our laboratory assays was inactive, indicating that at least some portion of unrecovered ticks in the wild were simply inactive at or near the site of release.

Numerous ecological and physiological factors are expected to affect tick movement and activity. These include abiotic conditions (e.g. temperature and humidity) [5, 61, 62], habitat structure (e.g. litter density) [26, 31, 46], and tick condition (e.g. age) [59, 63]. Weather conditions varied widely over the course of our experiments. We controlled for abiotic effects by including experiment date as a random effect in statistical models. This captured the multiple interacting abiotic conditions at the time of LST trapping. Experiment date affected the variation in tick recapture over time but had little effect on the distance of tick movement in the field. This suggests that weather may affect the proportion of the tick population that is detectable, but abiotic conditions may not strongly affect LST horizontal movement. Horizontal movement may be strongly affected by habitat structure (e.g. vegetation type and litter depth). Curtis et al. [14] observed that relative complexity of the vegetation and forest litter influenced movement of *Ixodes scapularis* over short distances (< 1 m). It was not feasible to accurately characterize micro-habitat structure in our experiments, given the range of LST movement covered up to 254 m^2^ of forest. Instead, we chose to include plot as a random factor in statistical models to capture variation in habitat complexity among replicates. We consistently observed that plot affected a large amount of variation in the distance LST moved and the proportion of LST recovered. This suggests that habitat complexity is an important determinant of LST movement and detection which warrants future examination.

We were not able to determine the ages of the ticks used in our studies, but it is likely that ages varied by months to over a year. Previous research has shown that many LST adults survive up to 2 years in the field [64], and we captured marked LST in 2024 that were released in 2023 (data not shown). Given the wide variation in timing of development and the long survival periods of adults, wild populations of adult LST likely represent a heterogeneous mix of ages [65]. In our experiments, we included date of initial tick collection as a random factor in statistical models to account for effects of tick age. We observed that date of collection was associated with a large degree of variance in tick movement and recovery (detection), suggesting that conditions of the ticks affected their behavior.

Host movement affects the spatial distribution of ticks [66–69] and provides the basis of host exclusion as a means of tick control [70]. In contrast, horizontal tick movement has been largely neglected in modeling approaches thus far, despite observations indicating this type of movement is involved with host acquisition and dispersal by several important tick species [14, 26, 28, 67]. Based on the marked ticks we captured in adjacent plots, we suspect that LST is drawn over long distances (tens of meters) towards host cues, which could impact the frequency at which LST contact their hosts. Inclusion of host movement and host-contact parameters have proven useful for predictive models of *Ixodes* spp. and *Amblyomma* spp. [11, 71–73] as have measures of intrinsic factors such as physiological age [63, 74] and water balance [11, 73]. Integration of tick movement and activity parameters such as we have presented here may improve precision of LST risk models and help focus mitigation efforts on the areas with the most impact.

Our results illustrate that LST are highly mobile and may control their spatial distributions independent of where they drop from previous hosts. The high mobility of adult LST makes them relatively fluid in the landscape and may affect the reliability of static point estimates for LST abundance. By including movement parameters, ecological models could more accurately predict distributions and densities of LST. Further exploration of the horizontal movement capabilities of medically important tick species and life stages is warranted for inclusion in population estimates and activity models. Importantly, we observed that approximately 30% of the adult LST population is detectable by CO_2_ trapping at any time, and undetected ticks can be recovered at later dates in the same geographic location. This suggests that undetected ticks are not absent but periodically inactive. The causes of this pattern remain to be determined, but the pattern is potentially useful for efforts to estimate LST abundance from surveillance with CO_2_ trapping. Our data reflect only activity and movement of adult LST. Juvenile life stages should be evaluated to better estimate entire tick populations as they may exhibit different behaviors than adults. Finally, additional information is needed on how adult LST navigates in different habitats and specifically how movement is impacted as a function of microhabitat complexity and climatic variables. Climate and habitat complexity could change LST movement and detectability. Thus, it will be important to explore effects of these factors in greater detail in the future. Further work is needed to address these knowledge gaps and should include field studies and modeling approaches.

## Conclusions

Understanding dispersal and detectability is important to our assessment of risk for contacting ticks and exposure to tick-borne pathogens [75]. We observed *A. americanum* ticks move up to 9 m across the forest floor in 24 h and can move up to 30 m after 72 h. Approximately 30% of the tick population was detected at any given time, and ticks remained detectable in locations for up to up to 14 days after release. This implies that a subset of the tick population is actively host seeking at any given time, and further study may provide investigators the ability to improve tick surveys and better estimate tick abundance. Given that risk models for tick contact are built around point estimates of tick activity in space and time, our data suggest that current models applied to LST may only be accurate for brief periods without accounting for tick spatial activity. The movement and activity patterns of LST should be incorporated into future modeling approaches for improved accuracy in estimating tick-host contact for this species.

## Supplementary Information

Supplementary material 1 Figure S1. Demonstration of marking combinations used for the abundance mark-recapture experiment. Figure S2. Image of laboratory behavior recording apparatus. Figure S3. Image of the dry ice traps used to recapture marked ticks. Figure S4. Map of central Oklahoma, USA. Figure S5. Map of Lake McMurtry, Stillwater, Oklahoma, USA. Figure S6. Map of mark-recapture sites at Lake McMurtry East Recreation Area, Stillwater, Oklahoma, USA.

Supplementary material 2 Table S1. The number of marked ticks released at timepoints prior to collection of the relative abundance experiment. Additional file 2_v2: Table S2. Model outputs for distance, time, and relative abundance experiments. Additional file 2_v2: Table S3. Distances and activity times of male and female *Amblyomma americanum *in the laboratory.

Supplementary material 3 Datasets of *Amblyomma americanum *mark-recapture distances and times, cardinal direction points, 30-m recaptures, relative abundances, and laboratory observations of distances traveled and activity times.

## Data Availability

All raw data are provided in the Additional file 3_v2.

## Abbreviation

LST: Lone star tick, *Amblyomma americanum*
