# Supplementary material for: Spatial and temporal activity patterns of Amblyomma americanum
Source: Parasit Vectors. 2025 Jan 16;18:12. doi: 10.1186/s13071-025-06661-x (PMC11740481; doi:10.1186/s13071-025-06661-x)

Figure S1. Image of unique marking combinations used in the abundance mark-recapture experiment. Designating one color for time and one for abundance allowed for simple, unique identifiers for ticks across time and plots. Photo credit: D.S. Marshall.

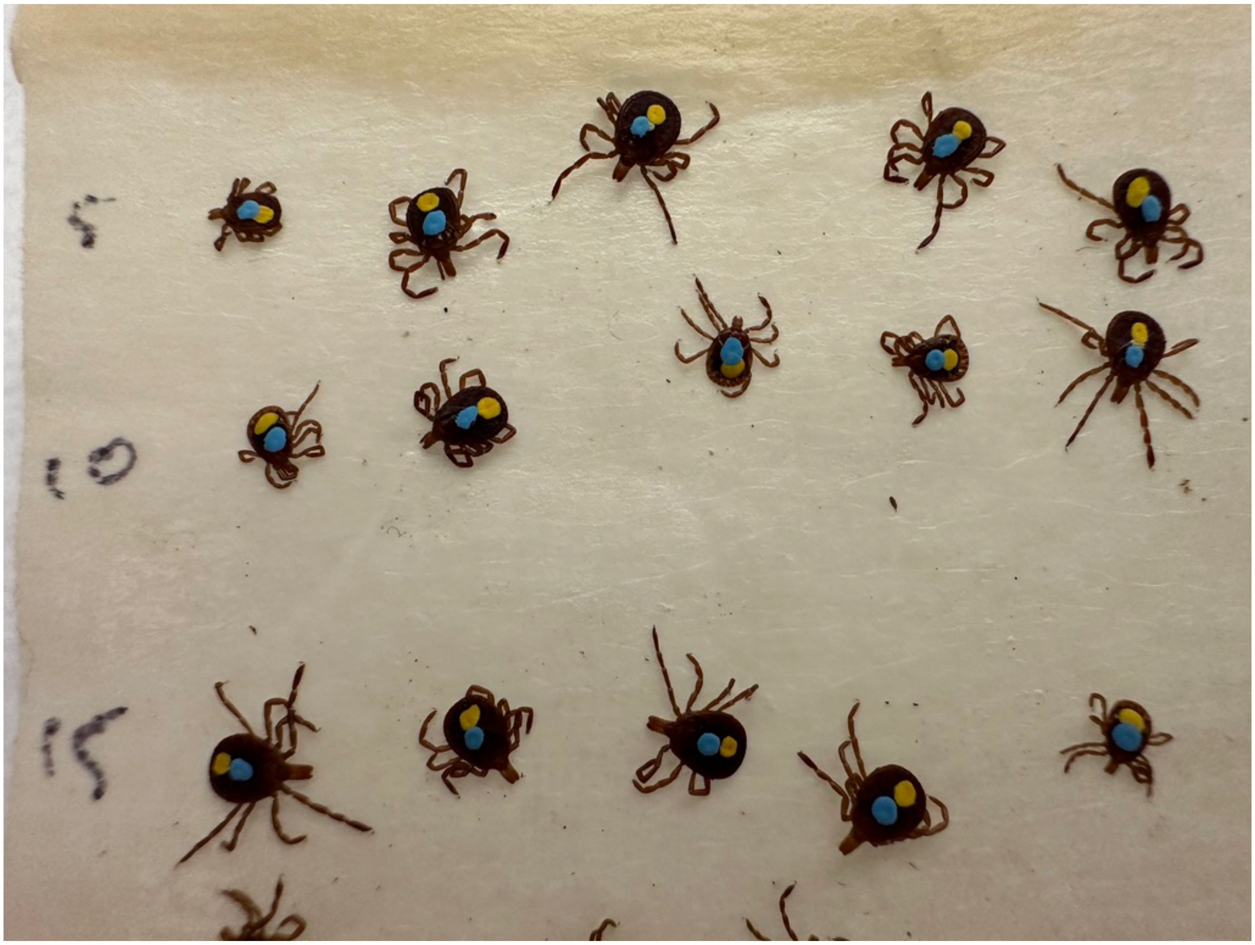

Figure S2. Image of our tick behavior set-up. Ticks were contained in petri dishes resting on an overturned aquarium. Inside the aquarium was a ring light with multiple sheets of mylar to diffuse light. The camera was mounted directly above the petri dishes so that ticks were lit from below to prevent glare. Photo credit: D.S. Marshall.

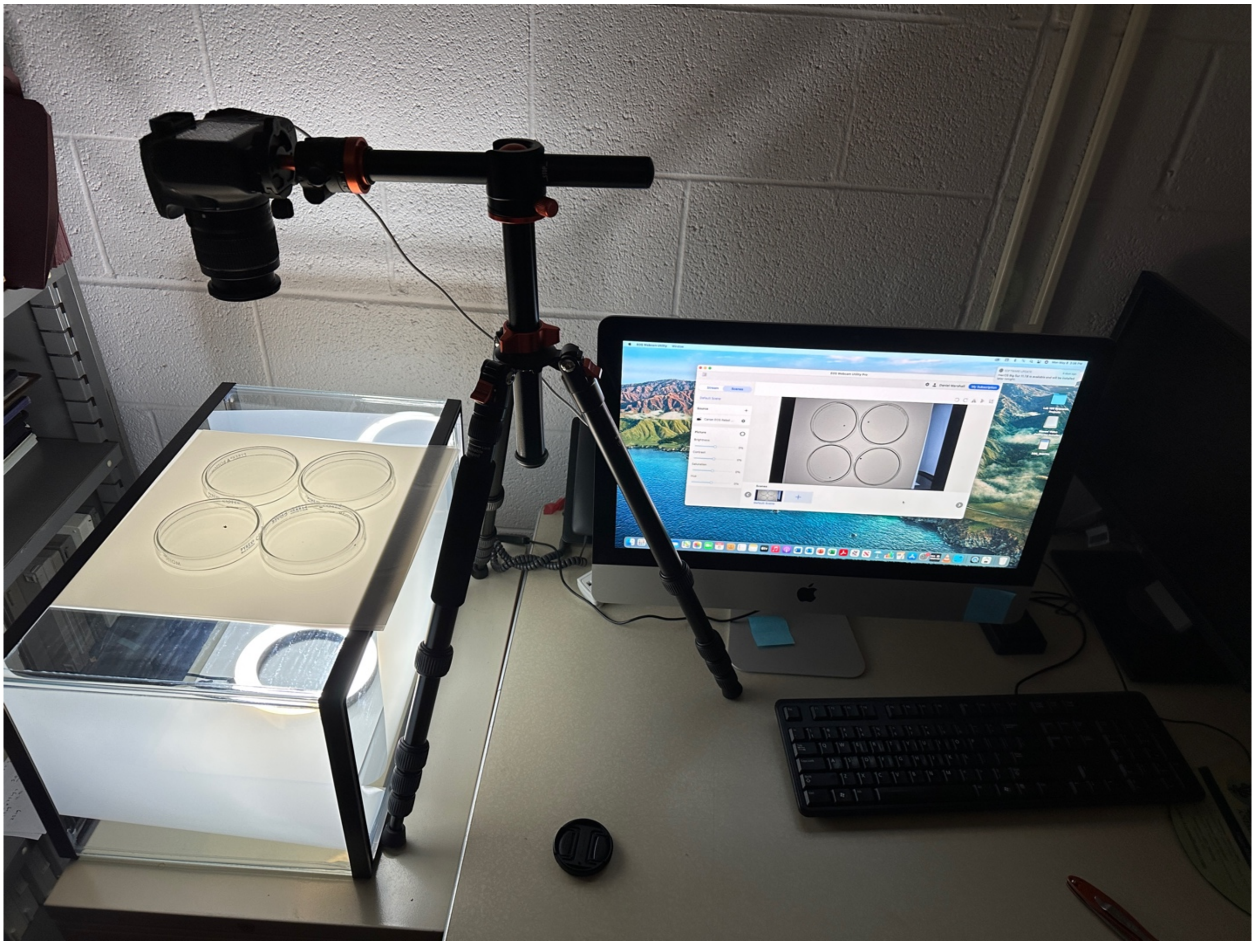

Figure S3. Photo of dry ice-baited trap. The trap is comprised of a 9 quart (8.5 L) Igloo brand cooler with eight, 0.635 cm holes drilled in the sides (three holes per long side, one per short side). Each trap contained approximately 2.7 kg of dry ice which sublimed through the holes.

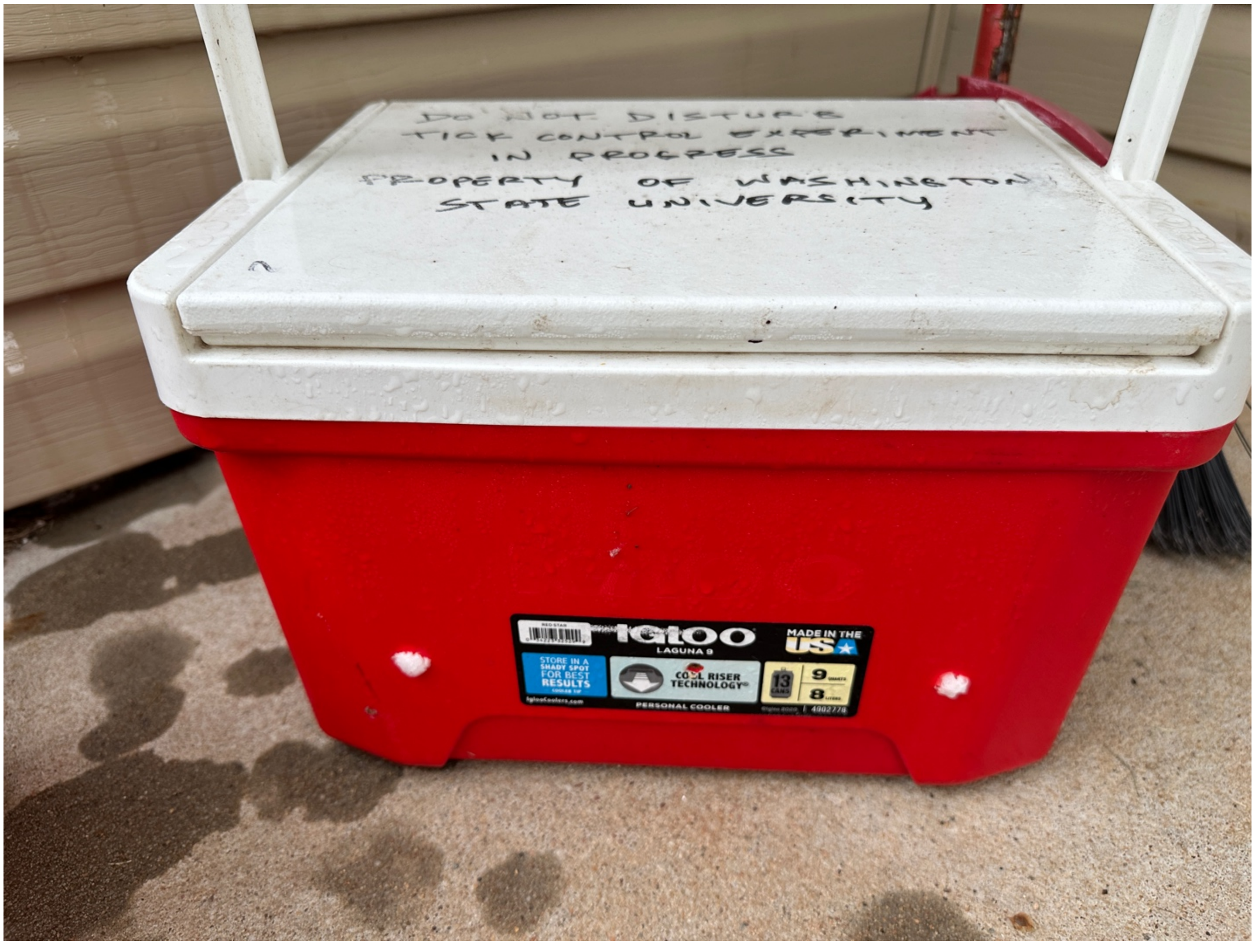

Figure S4. Map of central Oklahoma, USA. Our study site, Lake McMurtry is in north central Oklahoma near Stillwater. Map data: Google.

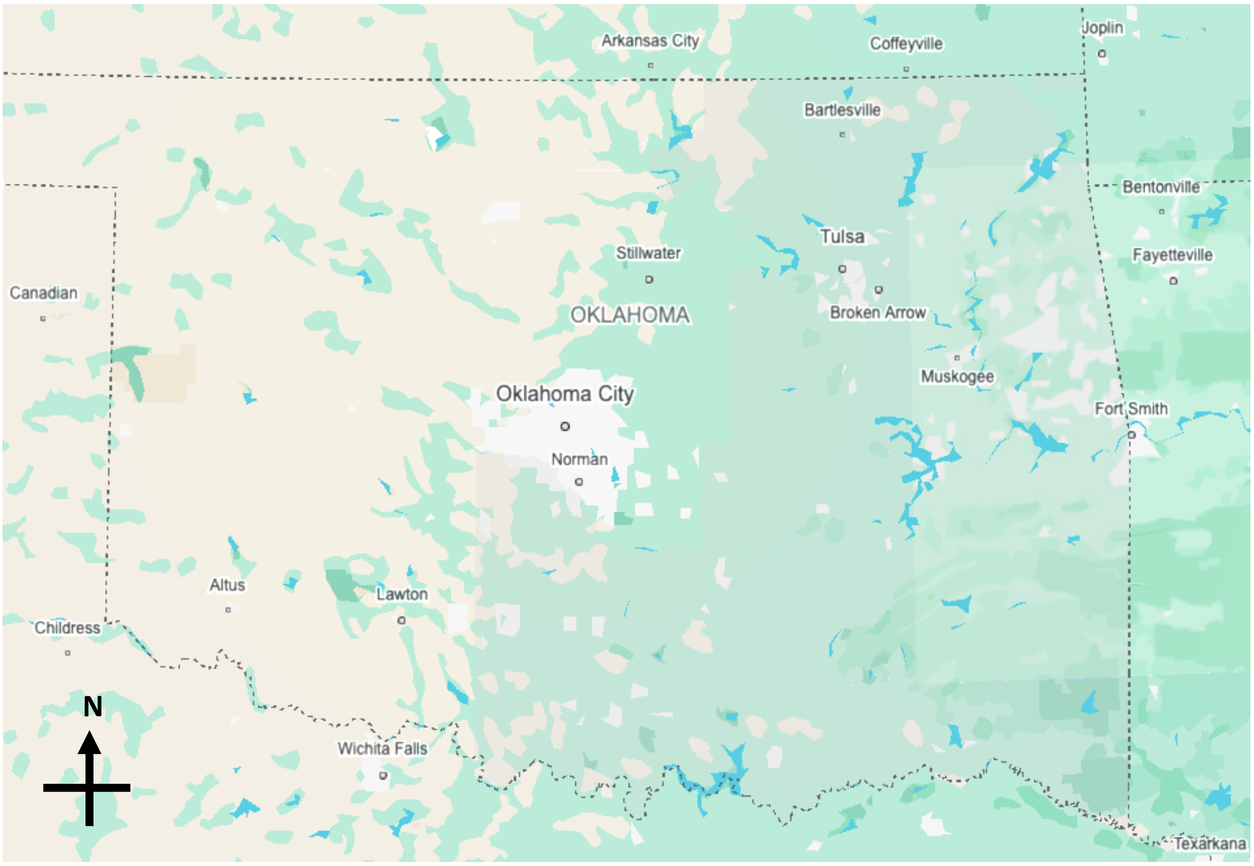

Figure S5. Map of Lake McMurtry, Stillwater, Oklahoma, USA. The yellow box indicates area of magnification in Figure S6. Map data: Google, Maxar Technologies.

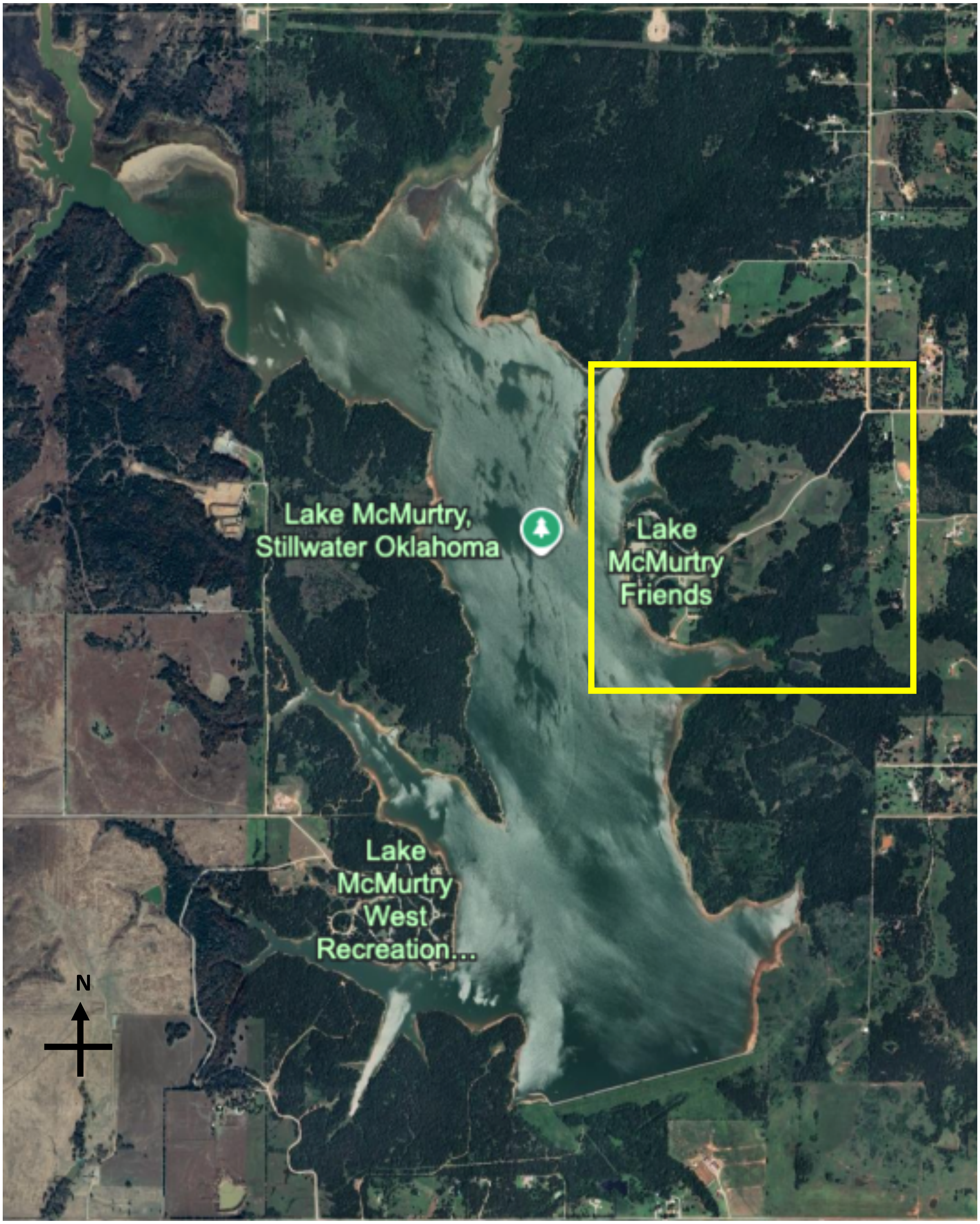

Figure S6. Map of Lake McMurtry East Recreation Area, Stillwater, Oklahoma, USA. Yellow polygons represent areas where mark-recapture experiments took place in 2023 and 2024. Map data: Google, Maxar Technologies.

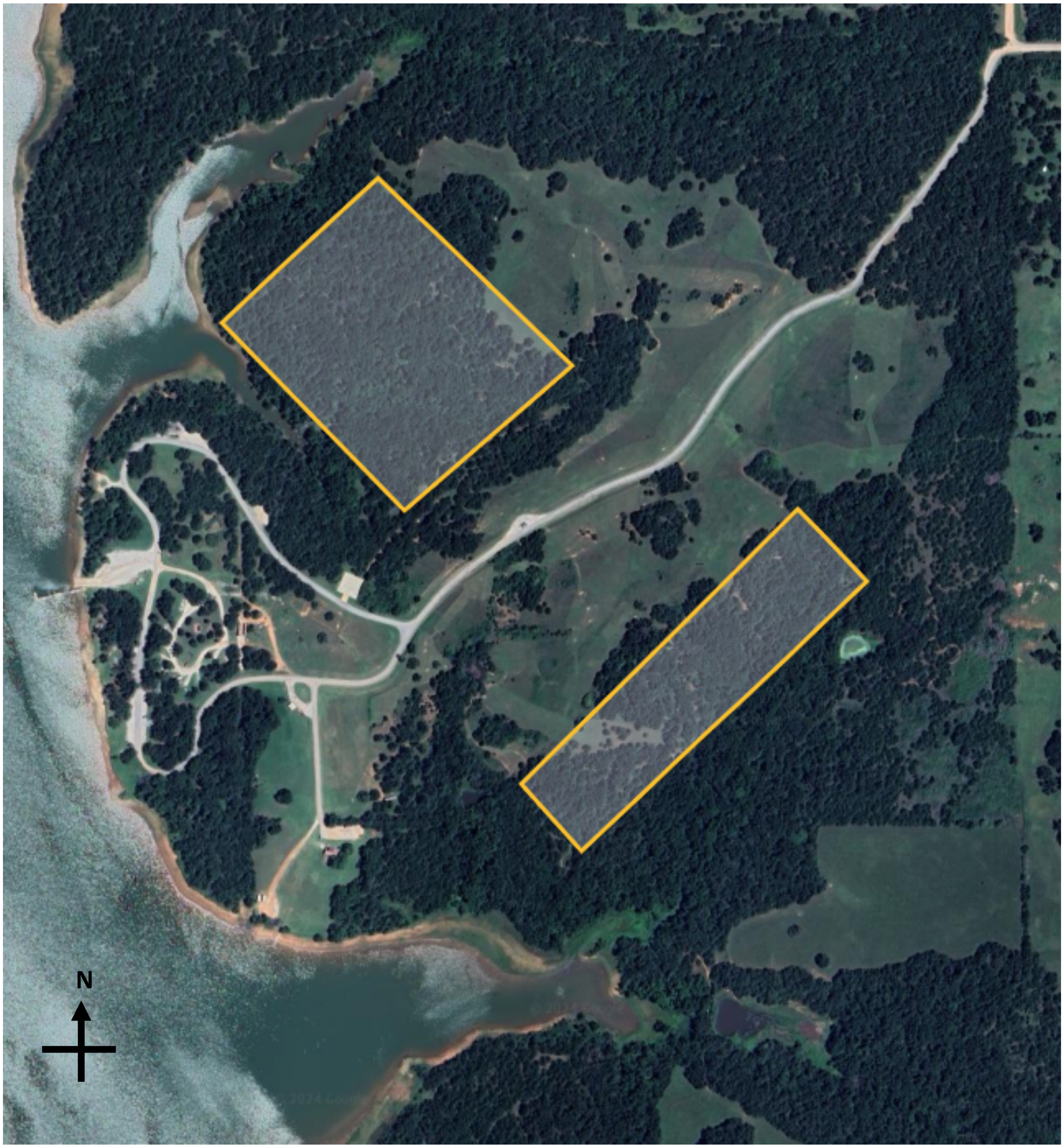

Supplement: Supplementary file 1 — Supplementary material 1 Figure S1. Demonstration of marking combinations used for the abundance mark-recapture experiment. Figure S2. Image of laboratory behavior recording apparatus. Figure S3. Image of the dry ice traps used to recapture marked ticks. Figure S4. Map of central Oklahoma, USA. Figure S5. Map of Lake McMurtry, Stillwater, Oklahoma, USA. Figure S6. Map of mark-recapture sites at Lake McMurtry East Recreation Area, Stillwater, Oklahoma, USA. [file 13071_2025_6661_MOESM1_ESM.pdf]
