# Supplementary material for: Spatial and temporal activity patterns of Amblyomma americanum
Source: Parasit Vectors. 2025 Jan 16;18:12. doi: 10.1186/s13071-025-06661-x (PMC11740481; doi:10.1186/s13071-025-06661-x)
Supplement: Supplementary file 2 — Supplementary material 2 Table S1. The number of marked ticks released at timepoints prior to collection of the relative abundance experiment. Additional file 2_v2: Table S2. Model outputs for distance, time, and relative abundance experiments. Additional file 2_v2: Table S3. Distances and activity times of male and female Amblyomma americanum in the laboratory. [file 13071_2025_6661_MOESM2_ESM.pdf]

Table S1. The number of marked ticks released at days 14, 7, and 3 before collection. Marked ticks were released in high relative abundance (60, 30, and 10 ticks) and low relative abundance (1 tick) batches. We used a full factorial design for the high abundance batches so that every density was replicated twice at each time point. Every high abundance release was accompanied by a uniquely marked single tick.

Plot number	14 days	7 days	3 days
1	60 + 1	30 + 1	10 + 1
2	30 + 1	60 + 1	10 + 1
3	60 + 1	10 + 1	30 + 1
4	10 + 1	60 + 1	30 + 1
5	10 + 1	30 + 1	60 + 1
6	30 + 1	10 + 1	60 + 1

Table S2. Table of distance, time, and relative abundance model outputs and parameters. The three models were used in our study are as follows:

Distance model: $Number_{marked} \sim Distance + (1|Date_{collection}) + (1|Date_{experiment}) + (1|Plot)$

Time model: $Recaptured\ Proportion_{\text{day X}} \sim Days + (1|Date_{collection}) + (1|Date_{experiment}) + (1|\ Plot)$

Relative abundance model: $Number_{marked} \sim Relative\ Abundance + Time + Relative\ Abundance * Time + (1|Date_{collection}) + (1|Plot)$)

		Random Effects		Fixed Effects			
Model	Predictor	Variance	Std. dev.	Estimate	Std. error	z-value	p-value
Distance model	Intercept			1.7997	0.3962	4.542	5.57E-6
	Distance			-0.1627	0.0332	-4.904	9.41E-7
	Plot	0.0962	0.3102				
	Date of capture	0.7644	0.8743				
	Date of experiment	3.914E-7	0.0006				
Time model	Intercept			-1.1409	0.3956	-2.884	0.0039
	Time (days)			-0.1853	0.0767	-2.418	0.0156
	Plot	0.1608	0.4010				
	Date of capture	0.2970	0.5449				
	Date of experiment	0.2664	0.5162				
Relative abundance model	Intercept			-0.2106	0.3805	-0.554	0.580
	Time (days)			0.0304	0.0384	0.791	0.429
	Relative abundance			0.0555	0.0081	6.879	6.03E-12
	Time x Relative abundance			-0.0009	0.0008	-1.057	0.290
	Plot	0.0179	0.1338				
	Date of capture	0.0267	0.1634				

Table S3. Distance and time spent moving in 24 hours by wild *Amblyomma americanum* in the laboratory.

Sex	Median distance (m)	Mean time spent moving (sd) (minutes)
Female (N=43)	29.4 (1.1–359.9)	550.6 (353.9)
Male (N=29)	36.1 (0.03–244.2)	530.8 (315.0)
Combined (N=72)	33.6 (0.03–359.9)	542.6 (336.6)
